# Construction and validation of BRAF mutation diagnostic model based on ultrasound examination and clinical features of patients with thyroid nodules

**DOI:** 10.3389/fgene.2022.973272

**Published:** 2022-09-08

**Authors:** Chan Xu, Jianqiang Fang, Wanying Li, Chenyu Sun, Yaru Li, Scott Lowe, Rachel Bentley, Shuya Chen, Cunyu He, Xinxin Li, Bing Wang, Chengliang Yin, Wenxian Li, Wenle Li

**Affiliations:** ^1^ Department of Dermatology, Xianyang Central Hospital, Xianyang, China; ^2^ Clinical Medical Research Center, Xianyang Central Hospital, Xianyang, China; ^3^ Department of Ultrasound Interventional, Xianyang Central Hospital, Xianyang, China; ^4^ AMITA Health Saint Joseph Hospital Chicago, Chicago, IL, United States; ^5^ Internal Medicine, Swedish Hospital, Chicago, IL, United States; ^6^ College of Osteopathic Medicine, Kansas City University, Kansas City, MO, United States; ^7^ Newham University Hospital, London, United Kingdom; ^8^ Faculty of Medicine, Macau University of Science and Technology, Macau, China; ^9^ Department of Orthopaedics II, The Second Affiliated Hospital of Xi’an Jiaotong University, Xi’an, China; ^10^ Center for Molecular Imaging and Translational Medicine, Xiamen University, Xiamen, China; ^11^ Beijing Life Biosciences Co., LTD, Beijing, China

**Keywords:** BRAF gene, thyroid nodule, nomogram, ultrasonography, prediction model

## Abstract

**Introduction:** Fine Needle Aspiration (FNA) is currently the most popular method for identifying benign and malignant thyroid nodules. However, its diagnostic sensitivity is sometimes limited, which makes it necessary to apply genetic testing and other modalities as a secondary diagnostic method. The diagnostic accuracy of thyroid nodule can be improved by combining mutations in the B-Raf proto-oncogene serine/threonine kinase (BRAF) with FNA. Thus, this study was conducted to create a nomogram diagnostic model based on the clinical and ultrasonic characteristics of patients with BRAF mutations to aid in the identification of benign and malignant thyroid nodules using FNA.

**Methods:** From April 2018 to December 2021, 275 patients with thyroid nodules who underwent ultrasonography and BRAF gene testing (137 positive and 138 negative) were included from Xianyang Central Hospital. The clinical and ultrasonic characteristics of the patients were used to develop a nomographic, diagnostic model of BRAF gene mutation, and to validate and evaluate the usefulness of the model.

**Results:** Independent risk factors for BRAF mutations included: focal strong echogenicity (microcalcifications, OR = 3.04, 95%CI = 1.41–6.58, *p* = 0.005), hypoechogenicity (OR = 3.8, 95%CI = 1.14–12.61, *p* = 0.029), lymph node metastases (OR = 3.54, 95%CI = 1.43–8.75, *p* = 0.006), margin (lobulated, OR = 3.7, 95%CI = 1.66–8.23, *p* = 0.001; extrathyroidal invasion, OR = 2.81, 95%CI = 1.11–7.06, *p* = 0.029), and shape (vertical position, OR = 2.7, 95%CI = 1.11–6.59, *p* = 0.029). The area under the curve (AUC) of the receiver operating characteristic (ROC) curve of the BRAF mutation diagnostic model constructed on these factors was 0.806 (95% CI = 0.754–0.851), and 39.5% was set as the threshold probability of making a clinical decision. The results of the validation and clinical utility evaluation showed that our model had good predictive performance and clinical application value.

**Conclusion:** Our nomogram diagnostic model based on clinical and ultrasound features of patients accurately predicted the possibility of BRAF gene mutations in patients with thyroid nodules.

## Introduction

Although the majority of thyroid nodules are benign, a small percentage are malignant. Papillary thyroid carcinoma (PTC) is the most common type of thyroid cancer, and its incidence has been on the rise in recent years (Miranda-Filho et al., 2021). With thyroid nodules being so common and the incidence of thyroid cancer on the rise, accurate determination of the benignity or malignancy of thyroid nodules is crucial to the choice of treatment (Li et al., 2022a). The use of Fine Needle Aspiration (FNA) cytology is currently the most common method of identifying benign and malignant thyroid nodules, and treatment measures such as persistent observation, repeat FNA, or surgery are often performed based on the initial diagnosis (Xing et al., 2013; Poller and Glaysher, 2017). However, FNA has the disadvantage of having a relatively low diagnostic sensitivity and requiring other modalities such as genetic testing to aid the diagnosis (Ahn, 2021; Holt, 2021).

Mutations in the B-Raf proto-oncogene serine/threonine kinase (BRAF) gene are the most common type of mutation in PTC and are almost absent in benign thyroid nodules (Wei et al., 2022). BRAF V600E is the main locus of BRAF mutations. Many studies have shown that the BRAF V600E mutation is strongly associated with the development and progression of PTC. As a highly effective risk-predicting and molecular marker for PTC, genetic testing for BRAF V600E mutation combined with FNA may improve the diagnostic accuracy of thyroid cancer and help surgeons to develop more individualized treatment plans (Kim et al., 2013a; Rashid et al., 2020). In addition, BRAF mutations are closely related to poor prognosis of tumors, and it is currently a hot topic of research on combining BRAF mutation testing and FNA to improve the accuracy of thyroid nodule diagnosis and effectively identify people at high risk of thyroid cancer (Poller and Glaysher, 2017; Goldner et al., 2019). However, the cost of BRAF gene testing is high, and there is a lack of diagnostic models for BRAF mutations, especially the nomogram based on simple clinical features and ultrasound findings of patients (Li et al., 2022b).

We attempted to develop a nomogram diagnostic model for BRAF mutations based on clinical and ultrasound features of patients to assist in the identification of benign and malignant thyroid nodules by FNA, thereby reducing clinical costs and improving diagnostic efficiency.

## Materials and methods

### Study subjects

A total of 275 patients (137 with BRAF gene mutations and 138 without mutations) with thyroid nodules who underwent ultrasound examination and BRAF gene testing were included in this study at Xianyang Central Hospital between April 2018 and December 2021. The patients’ clinical data and ultrasound reports of the nodules were retrospectively analyzed and the nodules were assessed using the American College of Radiology Thyroid Imaging Reporting and Data System (ACR TI-RADS) grading guidelines. 

The inclusion criteria were as follows: 1) the subjects were in good health and could tolerate the FNA; 2) the subjects had primary thyroid tumors; 3) the subjects had voluntarily signed an informed consent form for the FNA biopsy and BRAF mutation testing; 4) Patients had clear and complete ultrasound images of their thyroid nodules; 5) other clinical information was complete. Subjects who met all of the above criteria were included in this study.

Exclusion criteria were as follows: 1) uncooperative patients; 2) history of unexplained bleeding or a tendency to bleed; 3) unable to tolerate FNA due to underlying physical condition; 4) patients who refused BRAF mutation testing; 5) history of other malignancies; 6) incomplete ultrasound images or poor quality of ultrasound images; 7) other incomplete clinical information. Subjects meeting any of the above exclusion criteria were excluded from this study.

Ultrasound was performed and evaluated by two board-certified ultrasound radiologists with at least 5 years of experience in thyroid ultrasonography, and they reached consensus with each other through collaboration for any disagreement. The study was approved by the Ethics Committee of Xianyang Central Hospital (Number: 2022-IRB-68).

### Standardization of parameters

All parameters were standardized. Ultrasound signs included nodule composition (cystic or spongy = 0, mixed cystic = 1, solid = 2), echogenicity (no echogenicity = 0, high or isoechoic = 1, hypoechoic = 2 or very hypoechoic = 3), shape (horizontal = 0, vertical = 3), margin (smooth or blurred = 0, lobulated = 2, extrathyroidal invasion = 3), focal strong echogenicity (absent or large comet tail = 0, coarse calcification = 1, marginal calcification = 2, microcalcification = 3), elasticity (5-point elastography scale), anteroposterior dimension, transverse dimension, up and down dimension, and maximum dimension. Other information included age, sex (male = 1, female = 2), BRAF gene mutation (positive = 1, negative = 0), nodule location (left lobe = L, right lobe = R, isthmus = M), lymph node metastasis (no metastasis = 0, metastasis = 1), and whether it is Hashimoto’s thyroiditis (no = 0, yes = 1).

### Construction and validation of the model

Fifteen characteristics were selected for univariate and multivariate logistic regression analyses. The results of the multivariate logistic regression analysis were then combined to construct a nomogram.

The bootstrap sampling (200 times) was used for internal validation. The model was validated by plotting the receiver operating characteristic (ROC) curve to verify the efficacy and diagnostic value of the model, and obtaining the corresponding area under the curve (AUC) to verify the accuracy of the model. The ability of the model to predict BRAF mutations was verified by calibration plots.

### Evaluation of the model

The optimal threshold value is based on the value taken when the sensitivity and specificity of the ROC curve are at their optimal position. Based on this threshold value, patients were classified as having the mutated BRAF gene or unmutated BRAF gene. The risk density and clinical utility of the model were then evaluated and the corresponding threshold probabilities were obtained to assess the predictive ability of the model in distinguishing whether the BRAF gene was mutated or not. A decision curve analysis (DCA) compared the net benefit of the clinical application of the model with the value of the clinical application.

### Statistical analysis

The measurement data were expressed as mean ± standard deviation (SD), T-test was used to compare the measurement data between the two groups, and Chi-square test was used to compare the count data between the two groups. Logistic regression was used for univariate and multivariate analyses, and the best cut-off value was achieved by ROC curve analysis. R software (version 4.0.5) was used for statistical analysis and graphing. The “rms” package of R software was used to construct the nomogram, and medcalc was used to plot the ROC curves. R packages used for the development of this model included rms, foreign, caret, ggDCA, DynNom, regplot, caret, ggDCA, survival, survminer, plyr, DynNom, regplot, data. table, ggpubr, pROC, patchwork, and eoffice. *p* < 0.05 was considered a statistically significant difference.

## Results

### Baseline information and feature screening

**FIGURE 1:**
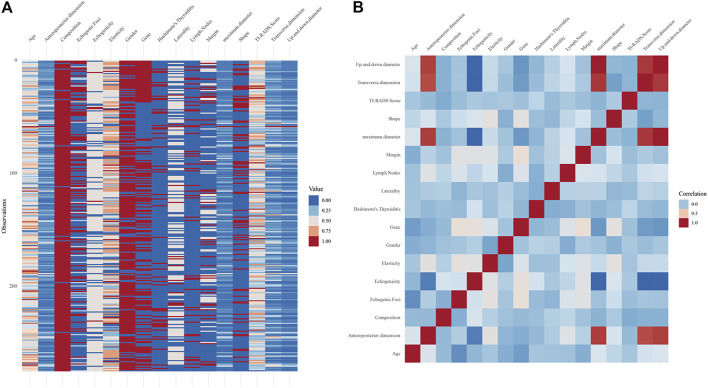
**(A)** Overview plot for all the included patients. **(B)** Heat map of the correlation for 17 characteristics.

All patients with thyroid nodules were enrolled between 2018 and 2021. Based on inclusion and exclusion criteria, a total of 275 patients (137 with BRAF gene mutations and 138 without mutations) were finally included in this study, including 58 men and 217 women, aged 17–80 years (mean age = 48.93 years). An overview of the data (Figure 1A) and a heat map of feature correlation (Figure 1B) were plotted using the 17 characteristics of the patients. For subsequent univariate and multivariate logistic regression analyses, 15 characteristics were screened.

### Univariate and multivariate logistic regression

Further univariate and multivariate logistic regression analyses were performed, and the odds ratio (OR) demonstrated the relative risk of BRAF gene mutations. Univariate logistic regression analysis showed that age (OR = 0.97, 95% CI = 0.95–0.99, *p* = 0.007), focal strong echogenicity (OR = 4.81, 95% CI = 2.63–8.79, *p* < 0.001), hypoechogenicity (OR = 9.48, 95% CI = 3.86–23.25, *p* < 0.001), elastography score of 3 or 4 (3, OR = 3.06, 95% CI = 1.34–6.99, *p* = 0.008; 4, OR = 3.46, 95% CI = 1.59–7.51, *p* = 0.002), lymph node metastasis (OR = 3.49, 95% CI = 1.68–7.26, *p* = 0.001), lobulated margin (OR = 6.74, 95% CI = 3.32–13.66, *p* < 0.001) and extra-thyroidal invasive margin (OR = 5.03, 95% CI = 2.3–11.02, *p* < 0.001), vertical position (OR = 3.8, 95% CI = 2.1–6.89, *p* < 0.001), maximum dimension (OR = 0.96, 95% CI = 0.93–0.99, *p* = 0.002), transverse dimension (OR = 0.94, 95% CI = 0.91–0.98, *p* = 0.004), and up and down dimension (OR = 0.96, 95% CI = 0.93–0.98, *p* = 0.001) were significant factors for BRAF mutations (Table 1).

**TABLE 1:** Univariate and multivariable logistics regression.

Characteristics	Univariate logistics regression			Multivariable logistics regression		
	OR	CI	P	OR	CI	P
Age	0.97	0.95–0.99	0.007	0.98	0.96–1.01	0.173
Anteroposterior.dimension	0.97	0.92–1.01	0.115	NA	NA	NA
Composition^a^						
1	Ref	Ref	Ref	Ref	Ref	Ref
2	0.66	0.18–2.38	0.522	NA	NA	NA
Echogenic.foci^b^						
0	Ref	Ref	Ref	Ref	Ref	Ref
1	0.93	0.41–2.1	0.863	0.54	0.2–1.44	0.220
2	1.52	0.09–24.78	0.768	1.07	0.06–20.01	0.965
3	4.81	2.63–8.79	<0.001	3.04	1.41–6.58	0.005
Echogenicity^c^						
0	Ref	Ref	Ref	Ref	Ref	Ref
2	9.48	3.86–23.25	<0.001	3.8	1.14–12.61	0.029
3	4.56	0.63–33.12	0.134	1.95	0.21–18.07	0.556
Elasticity^d^						
1	Ref	Ref	Ref	Ref	Ref	Ref
2	1.55	0.63–3.81	0.335	1.19	0.4–3.57	0.756
3	3.06	1.34–6.99	0.008	1.99	0.72–5.55	0.186
4	3.46	1.59–7.51	0.002	1.52	0.55–4.16	0.419
5	2.42	0.65–9.01	0.189	0.54	0.11–2.75	0.459
Gender						
Male	Ref	Ref	Ref	Ref	Ref	Ref
Female	1	0.56–1.79	1.000	NA	NA	NA
Hashimoto’s.thyroiditis						
No	Ref	Ref	Ref	Ref	Ref	Ref
Yes	0.73	0.39–1.38	0.333	NA	NA	NA
Laterality						
Left	Ref	Ref	Ref	Ref	Ref	Ref
Right	0.84	0.52–1.38	0.496	NA	NA	NA
Middle	1.05	0.36–3.08	0.935	NA	NA	NA
Lymph.nodes						
No	Ref	Ref	Ref	Ref	Ref	Ref
Yes	3.49	1.68–7.26	0.001	3.54	1.43–8.75	0.006
Margin^e^						
0	Ref	Ref	Ref	Ref	Ref	Ref
2	6.74	3.32–13.66	<0.001	3.7	1.66–8.23	0.001
3	5.03	2.3–11.02	<0.001	2.81	1.11–7.06	0.029
Maximum.diameter	0.96	0.93–0.99	0.002	1.24	0.79–1.95	0.357
Shape^f^						
0	Ref	Ref	Ref	Ref	Ref	Ref
3	3.8	2.1–6.89	<0.001	2.7	1.11–6.59	0.029
Transverse.dimension	0.94	0.91–0.98	0.004	1.11	0.95–1.29	0.200
Up.and.down.diameter	0.96	0.93–0.98	0.001	0.73	0.48–1.11	0.139

a Composition of nodules (mixed cystic = 1, solid = 2).

b Focal strong echogenicity (absent or large comet tail = 0, coarse calcification = 1, marginal calcification = 2, microcalcification = 3).

c Echogenicity (no echo = 0, hypoechoic = 2, very hypoechoic = 3).

d Elasticity (5-point scale for elasticity imaging).

e Margin (smooth or blurred = 0, lobulated = 2, extra-thyroidal invasion = 3).

f Shape (horizontal = 0, vertical = 3).

OR, odds ratio; CI, confidence interval.

Multivariate logistic regression analysis showed focal strong echogenicity (microcalcifications, OR = 3.04, 95% CI = 1.41–6.58, *p* = 0.005), hypoechogenicity (OR = 3.8, 95% CI = 1.14–12.61, *p* = 0.029), lymph node metastasis (OR = 3.54, 95% CI = 1.43–8.75, *p* = 0.006), margin (lobulated, OR = 3.7, 95% CI = 1.66–8.23, *p* = 0.001; extrathyroidal invasion, OR = 2.81, 95% CI = 1.11–7.06, *p* = 0.029), and shape (vertical position, OR = 2.7, 95% CI = 1.11–6.59, *p* = 0.029) were independent risk factors for BRAF mutations (Table 1).

### Construction and validation of the nomogram

**FIGURE 2:**
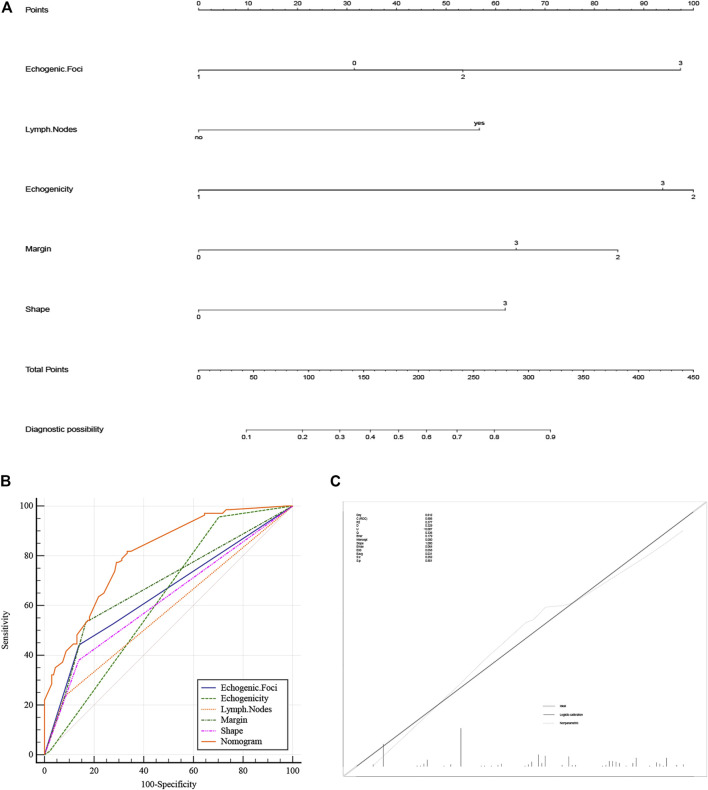
**(A)** Nomogram of the BRAF gene diagnosis. The corresponding node is selected on each variable axis and a straight line is plotted upwards to determine the score for each node. The sum of these numbers is positioned at the corresponding position on the total point axis and this line is plotted downwards to obtain the risk of BRAF gene mutation. **(B)** The model’s ROC curve. **(C)** Calibration plot showing the nomogram prediction and the actual observation. (The meanings represented by each node of strong echo, echo, edge and shape are the same as Table 1). ROC, Receiver Operator Characteristic.

A nomogram diagnostic model of the BRAF gene was constructed based on multivariate logistic regression results. We found that hypoechogenicity was highly associated with BRAF gene mutations, while lymph node metastasis was least associated with BRAF gene mutations (Figure 2A). ROC curves showed that the nomogram was more accurate for the diagnosis of BRAF gene mutation than univariate prediction (Figure 2B), with an AUC of 0.806 (95% CI = 0.754–0.851) (Table 2). Calibration plots showed a better fit between the model and the actual occurrence, i.e., the difference between the predicted and true situation of BRAF gene mutation (Figure 2C).

**TABLE 2:** Predict the AUC value of the model.

Variable	AUC	SE	95% CI
Echogenic.Foci	0.649	0.0290	0.590–0.706
Echogenicity	0.621	0.0227	0.561–0.679
Lymph.Nodes	0.577	0.0215	0.516–0.636
Margin	0.681	0.0273	0.622–0.736
Shape	0.621	0.0255	0.561–0.679
Nomogram	0.806	0.0254	0.754–0.851

AUC, area under curve; SE, standard error; CI, confidence interval.

### Clinical usefulness evaluation

**FIGURE 3:**
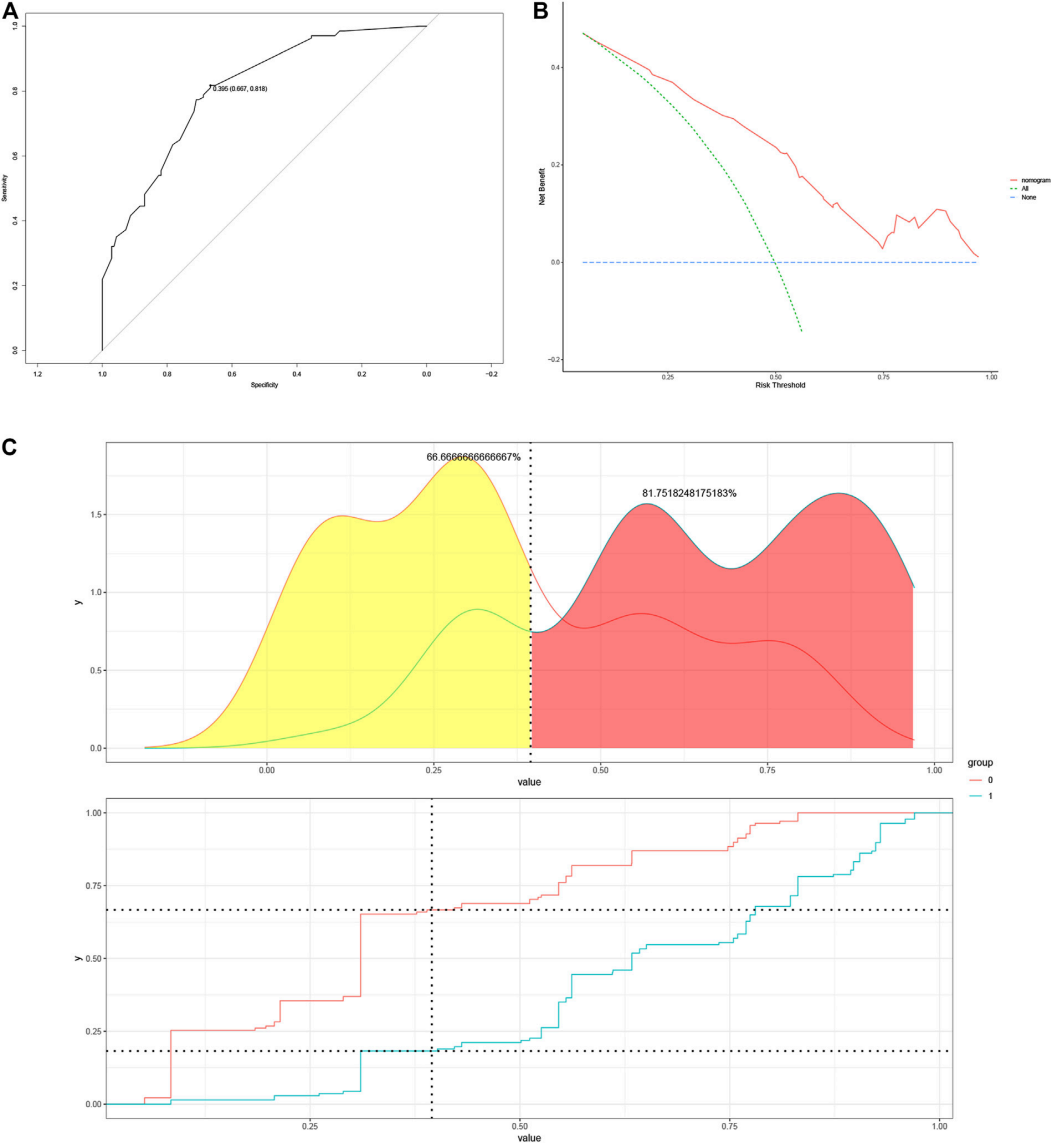
**(A)** Determination of optimal cut-off points based on ROC curves. **(B)** The risk density map and clinical utility map. The red curve represents BRAF gene without mutation, and the blue curve represents BRAF gene mutations. **(C)** DCA plots for the nomogram. ROC, Receiver Operating Characteristic; DCA, Decision Curve Analysis.

The optimal cut-off value was determined based on the maximum sensitivity (81.8%) and specificity (66.7%) of the ROC curve (Figure 3A). In addition, the mutated BRAF gene and the unmutated BRAF gene were grouped based on the optimal cut-off value. Risk density and clinical utility were plotted for the grouping (Figure 3B). The result indicated that the prediction model differentiated well between patients with BRAF gene mutations and patients without mutated BRAF genes, with high validity. The clinical utility plot showed the percentage of patients without BRAF gene mutations and patients with BRAF gene mutations detected at any probability threshold, and 39.5% was used as the threshold probability for making a clinical decision. The results of the DCA analysis showed a significant net gain of the diagnostic nomogram constructed in the present study, indicating a potentially good clinical effect of the model (Figure 3C).

## Discussion

The detection rate of thyroid nodules is increasing each year. Given that the treatment options for benign and malignant thyroid nodules vary considerably, early identification of benign and malignant thyroid nodules is critical. (Grani et al., 2020; Khan and Zeiger, 2020). Ultrasound and FNA are currently useful in the detection of thyroid nodules, but both have inherent limitations in their diagnostic capabilities. (Durante et al., 2018; Li et al., 2022c). The detection of mutations in the BRAF gene can assist in differentiating among thyroid nodules, but there are cost and time limitations associated with BRAF testing (Jinih et al., 2017; de Koster et al., 2018). Creating a risk assessment system for BRAF mutations based on simple ultrasound findings could significantly reduce healthcare costs and be convenient. The aim of this study was to develop a nomogram diagnostic model for BRAF gene mutations based on the ultrasound and clinical characteristics of patients.

In this study, independent risk factors for BRAF mutations were screened by multivariable logistic regression analysis, including microcalcifications, hypoechogenicity, lymph node metastases, lobulated margins, extra-thyroidal invasion, and vertical position (aspect ratio >1). Among these risk factors, hypoechogenicity was the strongest predictor of BRAF gene mutations.

BRAF has been studied extensively in thyroid FNA cytopathology. Its mutation rate can be as high as 80% in papillary thyroid carcinoma (PTC), but BRAF mutations are rarely seen in benign nodules. This makes BRAF as a unique tumor marker in PTC (Xing et al., 2013; Mayson and Haugen, 2019). Our study showed that the rate of BRAF mutations in patients with thyroid nodules alone was 49.82% (137/275). It is currently believed that the mutation of BRAF gene leads to the mutation of amino acids in the protein structure, which leads to the continuous activation of the corresponding kinase and activates the mitogen-activated protein kinase pathway, leading to carcinogenesis (Teng et al., 2017; Haroon Al Rasheed and Xu, 2019). PTC with the BRAF V600E mutation is very aggressive, prone to capsular invasion, lymph node metastases, and has a poor prognosis (Czarniecka et al., 2015; Liu et al., 2016). Our results also showed that BRAF mutations were highly associated with extra-thyroidal invasion and lymph node metastasis (Li et al., 2021).

Most of the current studies focus on the relationship between BRAF gene mutations and thyroid cancer, as well as factors related to prognosis, such as the relationship between BRAF mutations and thyroid cancer tumor invasion outside the perineum, lymph node metastasis, and pathological staging (Liu et al., 2014; Wang et al., 2016; Ma et al., 2020). There were few predictive models of BRAF gene mutations in thyroid nodules based on ultrasound as well as clinical parameters. More studies showed that microcalcification, lobulated margins, hypoechogenicity, and vertical position were significantly associated with ultrasound signs of thyroid cancer (Kwak et al., 2011; Kim et al., 2013b; Shi et al., 2021). As BRAF gene mutations were highly correlated with thyroid cancer, our results also showed that microcalcification, hypoechogenicity, lobulated margins, and vertical position were significantly associated with BRAF gene mutations.

There was some controversy regarding the correlation between BRAF mutations and ultrasound parameters (Li et al., 2022a). Most studies showed a correlation between BRAF mutations and ultrasound parameters. For instance, Kabaker et al. (2012) found that BRAF mutations were linked to the border of the thyroid nodule, the aspect ratio, and microcalcification. Sastre-Marcos (2015) reported that microcalcification and hypoechogenicity were found to be two independent factors correlated with BRAF mutations. Our results were consistent with their findings, with microcalcification, hypoechogenicity, lobulated margins, and an aspect ratio of greater than 1 being independent risk factors for BRAF mutations. However, it was also shown that BRAF V600E mutations were not associated with tumor size, tumor border, or calcification (Li et al., 2017).

Studies have shown that hypoechogenicity is an important ultrasound sign of suspected malignancy (Tessler et al., 2017). Our results showed that hypoechogenicity was the strongest predictor of BRAF mutation, with OR of 3.8 (95% CI = 1.14–12.61). It was suggested that the gene mutation was associated with ultrasound characteristics of increased aggressiveness of thyroid cancer, which was consistent with previous reports that BRAF mutation was associated with increased risk of adverse outcomes such as increased aggressiveness of thyroid cancer and disease progression (Xing et al., 2014; Yang et al., 2015). Lee et al. (2011) prospectively analyzed 991 thyroid nodules and found that extreme hypoechogenicity (OR = 30.744, 95% CI: 15.951–59.255) was most correlated with BRAF V600E mutation. Our results were fundamentally in line with theirs.

The increase in hardness of the thyroid cancer might result from the development of microcalcification, which occurred as a result of cellular malnutrition, degeneration, and eventually the formation of calcium salt deposits to form sand granules (Bai et al., 2009). Microcalcification was most commonly observed in malignant thyroid nodules and may be linked to BRAF gene mutations (Das, 2009). Our results also showed that microcalcification was the specific manifestation in thyroid nodules with BRAF mutations.

However, there are some limitations to this study. First, there might be selective bias. Second, the sample size was relatively small and no external validation was performed. Third, there might be differences in examination practices between physicians, which may lead to some bias in image evaluation. This suggests that large data and multi-center studies are needed in the future, as well as external validation to further improve the accuracy and usefulness of the model. Individualized management of thyroid cancer based on tumor risk stratification by genetic variation is now widely recognized, and molecular testing has evolved from single-gene BRAF testing to multi-locus analysis of multiple genes, such as gene fusions, single nucleotide polymorphisms, and copy number variants, suggesting that more in-depth multi-gene studies could be conducted in the future.

Despite some of the limitations, our study could help resolve the contradiction between the need for minimally invasive surgery and the need to accurately distinguish benign and malignant thyroid nodules. At the same time, our model could assist FNA detection and avoid the inherent diagnostic limitations of FNA such as uncertainty and false negative results. We expect our research to screen for low-risk thyroid nodules and high-risk thyroid nodules; to achieve more scientific and rational individualized management of thyroid nodules; and to establish a precise, standardized, and individualized management model.

## Conclusion

Our study used retrospective data collected from patients with thyroid nodules to develop a diagnostic nomogram for assessing the presence of mutations in the BRAF gene. The model was validated and evaluated, which revealed a good diagnostic value. To summarize, the prediction of BRAF gene mutations can significantly reduce the cost of genetic testing for the health care system and help guide clinicians in the individualized and accurate diagnosis and treatment of patients.

## Data Availability

The raw data supporting the conclusion of this article will be made available by the authors, without undue reservation.
